# Patient reports of health outcome for adults living with sickle cell disease: development and testing of the ASCQ-Me item banks

**DOI:** 10.1186/s12955-014-0125-0

**Published:** 2014-08-22

**Authors:** San D Keller, Manshu Yang, Marsha J Treadwell, Ellen M Werner, Kathryn L Hassell

**Affiliations:** Department of Health Policy and Research, Quality and Performance Measurement Program, American Institutes for Research, 100 Europa Drive, Suite 315, Chapel Hill, NC 27517-2357 USA; Children’s Hospital & Research Center Oakland, 747 52nd Street, Oakland, CA 94609 USA; The National Heart Lung and Blood Institute, 6701 Rockledge Drive MSC 7950, Bethesda, MD 20892-7950 USA; Division of Hematology, University of Colorado, 12700 E. 19th Avenue, Rm 9122, RC 2/MS B170, Aurora, CO 80045 USA

**Keywords:** ASCQ-Me, Computer adaptive testing, CAT, Item response theory, IRT, Patient-reported outcomes, PROs, Sickle cell disease, Validity(up to 10 allowed)

## Abstract

**Background:**

Providers and patients have called for improved understanding of the health care requirements of adults with sickle cell disease (SCD) and have identified the need for a systematic, reliable and valid method to document the patient-reported outcomes (PRO) of adult SCD care. To address this need, the Adult Sickle Cell Quality of Life Measurement System (ASCQ-Me) was designed to complement the Patient Reported Outcome Measurement Information System (PROMIS®). Here we describe methods and results of the psychometric evaluation of ASCQ-Me item banks (IBs).

**Methods:**

At seven geographically-disbursed clinics within the US, 556 patients responded to questions generated to assess cognitive, emotional, physical and social impacts of SCD. We evaluated the construct validity of the hypothesized domains using exploratory factor analysis (EFA), parallel analysis (PA), and bi-factor analysis (Item Response Theory Graded Response Model, IRT-GRM). We used IRT-GRM and the Wald method to identify bias in responses across gender and age. We used IRT and Cronbach’s alpha coefficient to evaluate the reliability of the IBs and then tested the ability of summary scores based on IRT calibrations to discriminate among tertiles of respondents defined by SCD severity.

**Results:**

Of the original 140 questions tested, we eliminated 48 that either did not form clean factors or provided biased measurement across subgroups defined by age and gender. Via EFA and PA, we identified three subfactors within physical impact: sleep, pain and stiffness impacts. Analysis of the resulting six item sets (sleep, pain, stiffness, cognitive, emotional and social impacts of SCD) supported their essential unidimensionality. With the exception of the cognitive impact IB, these item sets also were highly reliable across a broad range of values and highly significantly related to SCD disease severity.

**Conclusion:**

ASCQ-Me pain, sleep, stiffness, emotional and social SCD impact IBs demonstrated exceptional measurement properties using modern and classical psychometric methods of evaluation. Further development of the cognitive impact IB is required to improve its sensitivity to differences in SCD disease severity. Future research will evaluate the sensitivity of the ASCQ-Me IBs to change in SCD disease severity over time due to health interventions.

## Background

Owing to advances in therapy and increased use of preventative medicine, more than ninety-percent of individuals with sickle cell disease (SCD) now grow out of pediatric care. However their optimal functioning and well-being may be compromised by chronic comorbid conditions including multi-organ failure, pain and neurocognitive deficits [1-3]. Social and economic challenges are common, as well as barriers to accessing quality healthcare [1,4]. There is thus a need for skilled adult-oriented health care providers to deliver sickle cell care and a corresponding need to improve understanding of the long-term needs of adults with SCD.

Beginning in 2002, the National Heart, Lung and Blood Institute (NHLBI) conducted a series of conferences and workshops to determine ways to improve treatment for adults living with SCD [5]. Stakeholders communicated the need for a systematic, reliable and valid method for documenting adult patient-reported outcomes (PRO) of care. We developed the **A**dult **S**ickle **C**ell **Q**uality of Life **Me**asurement Information System, or ASCQ-Me (pronounced “Ask me”), to meet this need. In this article we detail the statistical methods used to identify the most precise measures to include in ASCQ-Me item banks and continue this introduction by summarizing the formative research upon which the current work was based (detailed in Treadwell, Hassell, Levine and Keller, 2013[6]).

### Formation of the conceptual framework for ASCQ-Me

Because the epidemiology and psychosocial functioning of adults with SCD is understudied and not well-documented, we conducted a comprehensive program of formative research including a systematic review of the literature. We also conducted detailed, structured interviews with 123 adults with SCD and 15 sickle cell experts who varied in geographic location, age and gender. On the basis of these data, we created an inclusive taxonomy of 140 life areas that were affected by SCD and summarized these in a model of relationships between the signs and symptoms of SCD and adult life experiences [6].

### Grounded item technique (GIT)

We “grounded” ASCQ-Me questions in actual events in the lives of the interview participants through a rigorous analytic protocol applied to the audio-taped group or individual interviews [7,8]. The content based on GIT was supplemented, when required, by questions based on legacy measures. For example, SCD providers and clinical investigators advocated for the importance of including questions on cognitive functioning as part of ASCQ-Me; yet no content related to attention, memory, language or problem solving emerged from the GIT interviews. So we based the draft cognitive functioning items for ASCQ-Me on the careful, thoroughly documented work of the Medical Outcomes Study [9].

ASCQ-Me questions were written in plain language comprehensible at the 6^th^ grade level or below. We avoided the use of clauses, and restricted the item content to one topic. To promote standardization, we used item formats comparable to the cross-National Institutes of Health (NIH) generic PRO measure development effort, the Patient-Reported Outcomes Measurement Information System (PROMIS®) [10]. Experts in cognitive testing from the Cognitive Laboratory at the American Institutes for Research reviewed all of the items to identify those that should be rewritten. Those that could not be rewritten without destroying the intended meaning were submitted to cognitive testing with patients and, if they were correctly understood, they were retained (otherwise they were discarded). The field test version of ASCQ-Me was based on this set of culled items.

The purpose of the ASCQ-Me field test data collection was to identify the ASCQ-Me items that would be the most precise measures of their constructs for inclusion in item banks suitable for administration using computer adaptive assessment software, determine appropriate scoring algorithms for those items, and evaluate the reliability and validity of scores based on those algorithms.

## Methods

### Participants

ASCQ-Me field test data were collected at seven geographically diverse sites of care with the assistance of site coordinators trained in a standardized study protocol. Four methods were used to recruit participants: some were invited to participate by personnel at each clinic; others responded to flyers posted in or near the clinics; some responded to a posting on the website for the Sickle Cell Disease Association of America; and still others were recruited by participants who had already taken the assessments. Prior to enrollment, potential participants were administered a short screener. To be eligible for the data collection, participants were required to be 18 years of age or older and to be diagnosed with sickle cell disease. People were excluded if they were younger than 18, did not have a diagnosis of SCD, had a diagnosis of sickle cell trait, or could not read English. Based on previous research findings [11], the targeted enrollment across sites was set to obtain sufficient sample size for the psychometric analyses (500 patients) assuming a ten-percent rate of no-shows. Thus we targeted 550 patients with diversity in terms of age and gender.

### Measures

Four item sets were developed: cognitive impact (28 items), emotional impact (28 items), social functioning impact (28 items) and physical impact (56 items). In addition, five items were developed for fixed format administration: pain episode severity (3 items) and frequency (2 items). Examples of the questions are presented in Table 1 below.

**Table 1:** **ASCQ-Me item sets (Health topics) and example questions**

**Topic**	**Example question**
Cognitive Impact	In the past 7 days, how often did you have trouble remembering things people had just told you?
Emotional Impact	In the past 7 days, how often were you very worried about needing to go to the hospital?
Physical Impact	In the past 7 days, how often were your joints very stiff during the day?
Social Impact	In the past 30 days, how much did your health make it hard for you to do things with your friends?
Pain Episodes	Using any number from 0 to 10, where 0 is no pain and 10 is the worst imaginable pain, how severe was your pain during your last pain attack (crisis)?

We sought to include a measure of SCD severity to evaluate the ability of ASCQ-Me measures to reflect differences in groups of people who differed in the extent of their disease. The challenge for us was that there is no consensus method for assessing SCD severity. SCD is characterized by the type of mutations to the pair of beta-hemoglobin (Hb) genes. Variations include Hb-SS, Hb-SC and Hb-Sβ [11,12] and individuals with Hb-SS usually, but not always, have more symptoms than those with other genotypes [13-15]. The variation of symptoms and sequelae within genotypes is so broad that genotype can not serve as a reliable indicator of disease severity [16-19]. Incidence and frequency of hospitalizations for vaso-occlusive incidents have been used as a marker of disease severity [20-23]; however, data indicate that a large percentage of patients who suffer from extreme pain never go to the hospital [24-26].

Nevertheless, adult sickle cell providers seeing a patient for the first time ask that patient a set of questions to gauge the severity of his or her disease. Blood transfusions and daily use of pain medicine are types of health care utilization associated with severity of SCD. Complications of SCD include asplenia, retinopathy, avascular necrosis, leg ulcers, kidney disease, stroke, and pulmonary hypertension. A medical history characterized by prescription pain medication, blood transfusions and a number of these diagnoses in a patient presenting with SCD could indicate severe disease [27-32]. In the absence of a consensus method for determining severity, we reasoned that a method which mimicked the clinical interview in content would identify patients who differed in the amount of SCD-related damage caused by their sickle cell and this could serve as a surrogate marker of disease severity.

Following the logic outlined above, we included a checklist of seven conditions usually secondary to SCD and two treatments indicative of severity as part of the data collection. For convenience, we refer to this indicator here as the SCD Medical History Checklist (SCD-MHC). Along with the checklist, we included 10 global health items from the PROMIS®, and 12 comorbidity checklist questions unrelated to SCD taken from the PROMIS® comorbidity questions (e.g. diabetes, pregnancy, hepatitis-C, HIV/Aids). We expected the SCD-MHC to be related to the PROMIS® globals as well as reported health care utilization and pain episodes; but, not to be related to the PROMIS® comorbidity questions which referred to conditions not associated with SCD.

### Data collection procedure

Patients signed a consent form after they arrived at one of seven geographically-dispersed sites of care participating in the ASCQ-Me field test. They were then seated at a computer and a site coordinator helped them to log onto the ASCQ-Me website. The coordinator then entered the SCD type of the respondent and assisted the respondent in reviewing a tutorial that demonstrated how to operate the mouse, select responses to questions, monitor progress toward completion, and take a break. Respondents proceeded to complete the survey on their own following the tutorial. The survey took about 55 minutes to complete on average. Respondents received an honorarium for their participation.

### Analytic methods

Prior to applying statistical models to the data, we evaluated data quality by calculating the percent of missing data for each question and flagging participants who responded in less than one second to any question (on the assumption that they were not reading the questions, see van der Linden & Krimpen-Stoop, 2003 [33]). We also evaluated the plausibility of the SCD-MHC as a measure of severity by examining its relationship to several variables which would be expected to be related to differences in health based on previous research including age, frequency and severity of vaso-occlusive episodes, frequency of emergency room visits, and the PROMIS® global ratings of health. The SCD-MHC was scored as the sum of the questions that were endorsed as has been the method employed in previous research with such checklists [33-36], and supported by research showing negligible differences between unit and alternative weighting methods for the scoring of checklists [37,38].

### Factor analysis

Within each of the four item sets (i.e., cognitive impact, emotional impact, physical impact, and social functioning impact), we conducted confirmatory factor analysis (CFA) using structural equation modeling (SEM) and followed common current practice with regard to indications of model fit [39,40]. We conducted exploratory factor analysis (EFA), with oblique rotation and identified the number of factors on the basis of a convergence among criteria such as the results of parallel analysis (PA) [41], a scree plot of the eigen values [42], number of factors required to explain the majority of the variance, and the simplicity of the factor pattern [43]. All factor analyses were conducted on polychoric correlation matrices and used maximum likelihood estimation.

### Multi-trait scaling analysis

The Multi-trait Analysis Program, [44,45] was used to: (1) calculate the correlation of an item with its scale, correcting for overlap [46], (2) compare that correlation to its correlation with all other scales in the analysis; (3) estimate the internal consistency reliability coefficients for each hypothesized scale; and (4) calculate the percentage of respondents with the highest and lowest scores possible, respectively, on each scale.

### Evaluation of unidimensionality

Most health question sets exhibit a certain amount of multidimensionality due to repeated use of common phrases within the question stem (e.g. “how many” or “how much”) or to content balancing within the health domain (e.g. including items targeting attention and memory within the cognitive functioning domain). Following Reise et al. (2007) [47], we considered an item set to be essentially unidimensional if any identified multidimensionality did not have consequences for the interpretation of the underlying concept (i.e. did not consequentially affect the relationship of items to theta) by conducting bi-factor analysis. We specified two models of the relationship of items to latent trait(s) but used Item Response Theory (IRT) Graded Response Models (GRMs) [48] instead of the SEM approach used by Reise and colleagues [47]. The unidimensional models specified a single latent trait and no correlated errors; whereas, each item was allowed to have a discrimination parameter on the general factor and one of the group factors (identified earlier by the parallel analysis and EFA) in the bi-factor model specification. Both the unidimensional and bi-factor IRT models were fitted using the IRT-PRO software [49]. As this was new software, we confirmed all analyses by conducting unidimensional and bi-factor CFA’s using the SAS CALIS procedure. Essential unidimensionality was supported if the Pearson correlation between the vectors of discrimination parameters under the unidimensional model and the bi-factor model was high (e.g. > 0.90) and the root mean squared difference of discrimination parameters between the two models was comparatively low [50].

### Item-level measurement bias

In an IRT framework, an item is defined as displaying measurement bias (i.e., differential item functioning) if the item response curves (i.e., item parameters) are not the same for the reference and focal group [51]. We implemented a rank-based strategy as proposed by Woods [52] to select anchor items, followed by the IRT-based Wald test method [53-55] (denoted here as IRT-Wald), to detect differential item functioning (DIF) for each item. The sample was homogenous with regard to race and the education level of respondents exceeded the question vocabulary (6^th^ grade level) so we did not evaluate DIF due to race or education; but we did evaluate DIF for age and gender.

### IRT calibration

We fit the GRM to estimate discrimination and location parameters for each item (using the marginal maximum likelihood method [56]) which, respectively, describe the strength of the item’s relationship to the latent trait and the position of the item on the trait continuum; and, together, determine the information function of the item.

### Validity

Evidence for the content validity of items and measures was provided by the GIT [7] and review of legacy measures [6]. The validity of each item as a measure of the underlying health domain was evaluated via CFA. In addition, we determined the ability of each measure to discriminate among levels of SCD severity indicated by the SCD-MHC.

## Results

### Respondent characteristics

At a total of 561 respondents, we exceeded our targeted number of 550 patients by 11. ASCQ-Me field test participants represented a range of ages and a mix of gender and genotype (see Table 2). During the past 12 months, one in five respondents reported two or more sickle cell related pain episodes and the majority indicated considerable suffering during these episodes. On a scale of 0 to 10 where 0 was no pain and 10 was the worst pain imaginable, the average rating was 8 and nearly 30% indicated that the pain was the “worst pain imaginable”. When asked to rate interference from pain during the last episode, 37% indicated needing help from family or friends or constant care from family, friends or health care providers. On the other hand, nearly one in three said that the pain caused minimal or no interference. The majority of respondents indicated that their most recent pain episode lasted more than a day, with nearly 50% saying that it lasted more than the better part of a week, and more than 20% suffered more than a week with their latest attack.

**Table 2:** **Characteristics of survey respondents**

**Characteristic**	**Percent of respondents**
**Age**	
18 to 24	29%
25 to 34	33%
35 to 44	20%
45 to 54	11%
55 to 64	6%
65 to 74	1%
**Gender**	
Male	36%
Female	64%
**SCD Type**	
Sickle Cell Anemia (SS)	64%
Sickle Hemoglobin C Disease (SC)	21%
Sickle Beta-Thalassemia Disease (Beta)	10%
Unspecified Sickle Cell Disease (Other)	5%
**How much did your last pain attack (crisis) interfere with your life?**	
I've never had a pain attack (crisis)	2%
Not at all, I did everything I usually do	10%
I had to cut down on some things I usually do	21%
I could not do most things I usually do	30%
I could not take care of myself…needed some help…	18%
I could not take care of myself and needed constant care…	19%
**About how long did your most recent pain attack (crisis) last?**	
I've never had a pain attack (crisis)	2%
Less than 1 hour	4%
1 - 12 hours	18%
13 - 24 hours	8%
1 - 3 days	22%
4 - 7 days	25%
1 - 2 weeks	14%
More than 2 weeks	7%
**Patient rating of severity of pain in last attack (0 to 10)**	
Mean	7.76
Standard Deviation	2.27

### Data quality

None of the participants was flagged for responding too quickly to the questions. Ten questions which addressed work functioning had high rates of missing data (>200 respondents), because a large portion of respondents were not employed. We were therefore not able to include these 10 questions in the analysis of the Social Functioning Impact measure.

To evaluate the suitability of the SCD-MHC as an index of SCD severity we examined its relationship to several variables which are related to differences in health including age [57], frequency and severity of vaso-occlusive incidents [58,25], frequency of emergency room visits [59,60] and the PROMIS® global ratings of health [61]. Table 3 shows the results of a series of general linear models in which the variables listed in the row headings were regressed onto the SCD-MHC or the checklist of 12 co-morbidities which are not secondary to SCD (pulled from the PROMIS® co-morbidity checklist). For convenience, we refer to the checklist of 12 co-morbidities that are not associated with SCD as the Non-SCD-MHC. The pattern of relationships described in Table 3 supports the validity of the SCD-MHC as an indicator of SCD severity for these patients because the relationships to other indicators of health are highly significant and consistent and cannot be explained on the basis of common method bias. As shown in Table 3, the SCD-MHC was related to four of five items measuring the reported frequency and severity of vaso-occlusive incidents, while the Non-SCD-MHC was significantly related to only one of five. The SCD-MHC was also significantly related to age and number of emergency room visits, while the Non-SCD-MHC was not. Finally, the relationship of the SCD-MHC to general health as measured by the 10 PROMIS® Globals was far stronger and more consistent than the relationship of the Non-SCD-MHC index to the 10 PROMIS® Globals.

**Table 3:** **Evaluation of the SCD-MHC as an indicator of SCD severity**

**Evaluation variables**	**F-Stat**	
	***SCD-MHC*** ^***ǂ***^	***Non-SCD-MHC*** ^***¥***^
What is your age?	47.07***	00.17
Past 12 mos, how many times did you go to the ED because of a pain attack (crisis)?	12.03***	03.00
**Severity of Vaso-Occlusive Events**	04.3*	00.80
Past 12 mos, how many sickle cell pain attacks (crises) did you have?	03.57	06.35**
When was your last pain attack (crisis)?	05.96*	00.27
Rate severity of pain in last attack	12.54***	00.03
How much did your last pain attack (crisis) interfere with your life?	13.24***	00.03
About how long did your most recent pain attack (crisis) last?	36.37***	03.10
**PROMIS Globals**	31.99***	02.29
In general, would you say your health is:	32.13***	02.29
In general, would you say your quality of life is:	10.22**	07.36**
In general, how would you rate your physical health?	10.64***	06.64**
In general, how would you rate your mental health, including your mood and your ability to think?	27.79***	18.82***
…, how would you rate your satisfaction with your social activities and relationships?	31.91***	07.80**
To what extent are you able to carry out your everyday physical activities?	6.59**	04.56*
Past 7 days, how would you rate your pain on average?	31.63***	00.61
Past 7 days, how would you rate your fatigue on average?	03.07	10.13**
Past 7 days, please rate how well you carry out your usual social activities and roles	47.07***	00.17
Past 7 days, how often … bothered by emotional problems such as…?	12.03***	03.00

^ǂ^SCD-MHC = Sickle Cell Disease Medical History Checklist.

^¥^Non-SCD-MHC = Checklist of medical history conditions not associated with Sickle Cell Disease.

### Item bank construction

#### Unidimensionality

Although respondents were allowed to skip any of the field test questions, only five respondents had to be eliminated from the psychometric analysis due to missing data. This represented just one percent of the total number of respondents. Thus the psychometric analysis was conducted using 556 respondents. Item-total correlations for the Physical Impact item set confirmed our suspicion that the content of that bank would more appropriately reside in three groups representing pain (17 items), stiffness (19 items) and sleep functioning (20 items). Going forward, we evaluated the pain, stiffness and sleep functioning questions as three distinct item sets. This resulted in six item sets in total. Items were eliminated from three of the six due to low item-total correlations (see Table 4, 1st column under "causes for elimination").

**Table 4:** **Number of items eliminated and cause for elimination**

		**Cause for elimination**				
**Question set**	**Original # of items**	**Item-total correlation < 0.40**	**Simple structure violation**	**Local dependence**	**DIF**	**Final # of items**
Cognitive	28	5	3	5	0	15
Emotional	28	5	1	0	2	20
Pain	17	0	0	4	0	13
Sleep	20	2	0	4	2	12
Social	28	3	1	3	4	17
Stiffness	19	0	1	2	1	15

EFA, restricted to the number of factors that emerged in the PA, were conducted on the remaining items in each question set to identify the simple structure pattern that would be modeled by the IRT bi-factor analysis. Items which did not conform to simple structure were eliminated from four of six question sets (see Table 4, 2nd column under "causes for elimination"). The subsequent IRT analysis identified local dependence among pairs of the remaining items in five of six question sets. One item from each pair was deleted from the sets (see Table 4, 3rd column under "causes for elimination").

#### DIF

A total of nine items from four of the question sets demonstrated measurement bias with regard to age or gender and so were eliminated (see Table 4, 4th column under "causes for elimination" and Table 5). Table 5 lists the items for which we found DIF due to gender or age following the methods detailed above.

**Table 5:** **Items with biased measurement across genders or ages**

**Question set**	**Gender bias**	**Age bias**
**Emotional**		… feel very good about your health?
		… feel very hopeful about your health?
**Sleep**	… if you woke… how easy ….to fall back asleep?	
	… how easy …to fall asleep?	
**Social**		…your family…worried about your health?
		…health make it hard for you to go places?
	…have to change the plans because of your health?	…have to change the plans because of your health?
**Stiffness**		… impossible for you to move…legs or arms

### Reliability

Reliability statistics for ASCQ-Me are presented in the first two columns after the row headings of Table 6 which show, respectively: (1) the range of scores (out of a possible range of 6) wherein measurement error is below the threshold that is associated with greater than 90% systematic or predictable variance based on the underlying construct (based on information curves produced by the IRT 2-parameter GRM); and (2) Cronbach’s alpha coefficient estimate of internal consistency reliability [62]. The first column shows highly reliable measurement in the range of 4–5 out of 6 of the possible score distribution. We do not expect highly reliable measurement in the tails of the score range, by definition, as restricted range decreases statistical power. The second column shows the average reliability of scores for the measures to be well above the recommended 0.90 for use at the individual-patient level.

**Table 6:** **Reliability and validity of ASCQ-Me item banks**

**Question sets**	**Score range at < 0.33 SE** ^**§**^	**Internal consistency reliability**	**Model fit** ^**ψ**^		Corr. between models*	Relat. to SCD severity (F-stat**)
			**CFI** ^**¥**^	**RMSEA** ^**ǂ**^		
**Cognitive Impact**	3.7	0.95	0.95	0.10	0.96	02.92
**Emotional Impact**	4.1	0.96	0.93	0.09	0.97	16.56
**Pain**	4.0	0.95	0.94	0.09	0.96	31.67
**Sleep Impact**	4.1	0.92	0.98	0.08	0.96	12.13
**Social Impact**	4.8	0.95	0.95	0.10	0.96	20.51
**Stiffness**	4.4	0.95	0.94	0.11	0.96	38.72

^§^This is out of a total scale score range of 6 which includes those at the very top and bottom of the range.

^ψ^Confirmatory Factor Analysis was conducted using structural equation modeling in which the responses to each question were modeled as comprised of the influence of a single dimension and a number of group factors.

^¥^Comparative fit index (CFI) compares the variance explained by the model to the total amount of variance in the data. The CFA runs from “0” (no relationship between the predicted and observed correlation matrix) to “1.0” (the predicted correlation matrix is identical to the observed).

^ǂ^Root Mean Square Error of Approximation (RMSEA) is the amount of variance that is not predicted by the model.

*These are the correlations between the vector of discrimination parameters for the bi-factor with the uni-factor Item Response Theory Graded Response Model (IRT GRM) which indicate the amount of agreement between the two models in the relationship of items to the underlying construct.

**All F-statistics were significant at p < 0.0001 with the exception of that associated with the cognitive functioning measure which was > 0.05.

### Validity

The third column of Table 6 displays statistics for bi-factor models of the relationship of items to a single underlying dimension with secondary dimensions modeling artifactual covariances for each ASCQ-Me item bank. All comparative fit indices (CFI’s) are above the liberal criterion for good model fit (0.90) and three of six are at or above the more conservative criterion of 0.95. While the root-mean-square error of approximation (RMSEA) rates are higher than the range of 0.06-0.10 that is typically recommended in psychometric texts, [39,63] they are consistent with that reported in the literature for other PRO measures of this type [64] and with findings regarding the relationship of fit indices to degrees of freedom [65].

The next to the last column in Table 6 shows the correlations between the vectors of discrimination parameters for the bi-factor and uni-factor IRT models exceed 0.95 in every case. This suggests that the secondary factors which were modeled, dealt with sources of variability unrelated to the primary factor.

The last column in Table 6 displays additional evidence for the validity of the ASCQ-Me measures. We looked at the relationship of ASCQ-Me scores to the alternative marker of SCD condition severity based on the number of SCD sequelae and treatments endorsed from a list of nine, total (that is, the SCD-MHC). We divided respondents into tertiles based on the distribution of the SCD-MHC and tested the significance of the difference in ASCQ-Me scores across these tertiles (F-statistic from univariate analysis of variance models). The ASCQ-Me scores for all measures with the exception of the cognitive functioning measure, significantly differed according to SCD severity level (p < 0.0001), such that those with the least severe disease had the highest scores (demonstrating better patient-reported outcomes).

## Discussion

### Summary

We evaluated the reliability and validity of 140 questions designed to provide data that could be used to measure the patient-reported functioning and well-being of adults with SCD by conducting psychometric analysis of data from 556 patients. These patients, varying in age, gender, SCD Type, and SCD severity, provided us with high-quality responses to questions administered through the internet in a range of clinical settings. We eliminated 48 questions that either did not form clean factors or provided biased measurement across subgroups defined by age and gender. As a result, we derived six item banks that measure cognitive, emotional and social functioning, sleep quality, pain and stiffness. These six measures, collectively called ASCQ-Me, provide highly reliable measurement according to IRT total information curves as well as internal consistency reliability estimates. Analysis of construct validity supported the essential unidimensionality of the item banks and the measures discriminate among levels of disease severity defined on the basis of medical history. With one exception, across multiple criteria, we recommend the use of these item banks to contribute to studies of the health of patients with SCD. We do not recommend the use of the Cognitive Impact measure at this time because, compared to the other measures, it provided reliable measurement across a more restricted score range and was far weaker in discriminating among levels of severity in SCD.

The psychometric analyses detailed here were used to identify a final set of questions which produce reliable health outcome scores using far fewer than the 140 questions to which patients in the field test responded. As shown in Table 4, the five recommended item banks (absent the cognitive bank) include just 77 items, following elimination of questions based on the psychometric analysis. Moreover, the purpose of constructing the IRT-calibrated item banks was to identify still smaller subsets of items within each that can be used to provide precise measurement for particular applications. IRT calibrations indicate which items are most informative at a particular level of the trait being measured and thus enable the construction of short form measures. For example, we have constructed reliable short form measures totaling just 5 questions for each of the five recommended item banks. This enables users to measure all five ASCQ-Me concepts using just 25 questions. Moreover, IRT-calibrated item banks such as those included in ASCQ-Me can be administered using computer adaptive software the purpose of which is an alternative way to produce reliable measurement with as few questions as possible. Such software has been developed for ASCQ-Me.

### Limitations

We were limited by the study design in the range of analyses we could do to address the validity of the item banks. Data were collected at one point in time so we could not address the relationship of ASCQ-Me scores to change in condition. We had one clinical indicator of disease and this is known to be a poor measure of severity. Previous research supports the validity of self-report methods of multi-morbidity assessment, [66] and so we put considerable thought and careful analysis into developing the self-reported medical history checklist (SCD-MHC). We described the relationship of checklist scores to other markers of health burden: age, utilization, pain episode recency and severity, and PROMIS® global ratings of health. Because they derive from the same source, the relationship between SCD-MHC scores and ASCQ-Me item bank scores might be artifacts of the data collection method. This hypothesis was not supported because the ASCQ-Me scores did not have a strong and consistent relationship to a comorbidity index that was comprised of self-reported conditions which are not characteristic of SCD (e.g. migraine, cancer, rheumatoid arthritis).

The respondents included a mix of ages, gender, and disease type however those older than 54 (7%) and with SCD Type other than SS were in the minority. This prevented us from conducting psychometric analyses specific to patients with SC (21%) or Beta types (10%) or who were older than middle aged.

We do not know how representative our field test sample is because a nationally-representative, descriptive study of the socio-demographic and health characteristics (e.g. severity of disease, incidence of comorbid conditions) of adults with SCD does not yet exist. Such a study is hampered by the difficulty in developing a comprehensive sampling frame. That frame cannot rely on medical records or registries alone because many adults with SCD are not included in those data bases. Moreover, the stigma associated with SCD is a barrier to accurately identifying the names and contact information of individuals. However, this issue is not unique to the current research and indeed applies to all research results involving adults with SCD which seek to generalize to the population. Current scoring for the ASCQ-Me item banks is relative to this field test sample so that a score of 50 represents the average score for the 556 respondents. Ideally, we would like to be able to say that a score of 50 represents the average response of all adults with SCD. While we do not have a descriptive study of adults with SCD, available data suggests that the characteristics of our sample are likely to mirror those of the other populations with regard to age of participating adults and hemoglobin type [67-69]. Adult males with SCD may have been under-represented in our sample. Although results have been mixed [70] female gender has previously been associated with diminished health-related quality of life in the physical domain [68] and reports of a higher prevalence of pain episodes [6]. Our results should therefore be viewed with caution, until such time as a population-based description of socio-demographic and health characteristics of the U.S. SCD population is available.

### Future research

Further analyses of the field test data will be conducted to evaluate the potential of identifying cut-off scores for the ASCQ-Me item banks and short forms. Such scores could serve as interpretative aids which would enhance the usefulness of the ASCQ-Me measures for clinical practice.

While sample size prevented us from including the work-functioning questions in the IRT analysis conducted to develop ASCQ-Me, we intend to explore the development of a static work functioning scale for adults with SCD based on the field test items and data. Employment and work functioning were of great concern to participants in our focus groups and these concerns frequently surfaced as well in our individual interviews with adult patients.

Finally, should resources become available, we intend to collect longitudinal data with the ASCQ-Me measures which would permit us to evaluate the sensitivity of these measures to change over time. An ideal study would be a placebo-controlled study in which the size of the change in ASCQ-Me scores associated with the introduction of a treatment of known efficacy was evaluated. We are aware, also, of ongoing studies conducted by other investigators in which ASCQ-Me data are being collected longitudinally to evaluate the efficacy of drug therapy and to evaluate the impact of differences in health care delivery systems, and eagerly anticipate their reports.

## Conclusions

A valid measure of health outcome is required to inform the design and delivery of health care for adults with SCD. Building on a comprehensive program of formative research and statistical analysis of field test data on more than 550 patients, we applied advanced psychometric methods including those currently used by the PROMIS® initiative [10,71] to construct the ASCQ-Me measures of Cognitive, Emotional, Pain, Sleep, Social and Stiffness Impact. Our results support the reliability and validity of all measures, with the exception of Cognitive Impact, and we encourage the use of the remaining five measures in future studies conducted by the broader research community.

The contribution of research described herein was to develop a system called ASCQ-Me to provide a standard method for describing the life impact of SCD on adult functioning and wellbeing which would enable the comparison of health outcomes for these patients across medical, clinical and health services research on SCD. Strengths of this research include: 1) roots in a rigorous program of formative research with adults with SCD [6]; 2) the participation of a large number of adults with SCD in the field test (>550 patients), 3) the application of advanced psychometric methods consistent with standards put forth by the PROMIS® initiative [10,71], 4) a careful focus on evaluating and eliminating sources of bias in measurement, 5) evaluation of the validity of the ASCQ-Me measures using a measure of SCD severity that does not suffer from the weaknesses of often-used measures such as number of hospitalizations or SCD type, and 6) the development of item banks which can support the construction of short sets of questions for each health concept suitable for administration via fixed forms or adaptively, to provide precise measurement with as few questions as possible. This system is currently in use in a number of studies which will provide further information on the validity of the scores and the usefulness of the system; including studies of the sensitivity of ASCQ-Me scores to change in health that might result from drug therapy or from changes in how health care is delivered.

## Abbreviations

ASCQ-Me: Adult sickle cell disease quality of life measurement information system
CAT: Computer adaptive testing
CFA: Confirmatory factor analysis
DIF: Differential item functioning
EFA: Exploratory factor analysis
GIT: Grounded item technique
GRM: Graded response model
IRT: Item response theory
NHLBI: National heart, lung and blood institute
NIH: National institutes of health
PA: Parallel Analysis
PRO: Patient-reported outcome
PROMIS: Patient-reported outcome measurement information system
RMSEA: Root mean square error of approximation
SCD: Sickle cell disease
SCD-MHC: Sickle cell disease medical history checklist
SEM: Structural equation modeling

## References

[1] Brawley OW, Cornelius LJ, Edwards LR, Gamble VN, Green BL, Inturrisi C, James AH, Laraque D, Mendez M, Montoya CJ, Pollock BH, Robinson L, Scholnik AP, Schori M (2008). National institutes of health consensus development conference statement: hydroxyurea treatment for sickle cell disease. Ann Intern Med.

[2] Vichinsky EP, Neumayr LD, Gold JI, Weiner MW, Rule RR, Truran D, Kasten J, Eggleston B, Kesler K, McMahon L, Orringer EP, Harrington T, Kalinyak K, De Castro LM, Kutlar A, Rutherford CJ, Johnson C, Bessman JD, Jordan LB, Armstrong FD, Neuropsychological Dysfunction and Neuroimaging Adult Sickle Cell Anemia Study Group (2010). Neuropsychological dysfunction and neuroimaging abnormalities in neurologically intact adults with sickle cell anemia. JAMA.

[3] Claster S, Vichinsky EP (2003). Managing sickle cell disease. BMJ.

[4] Haywood C, Beach MC, Lanzkron S, Strouse JJ, Wilson R, Park H, Witkop C, Bass EB, Segal JB (2009). A systematic review of barriers and interventions to improve appropriate use of therapies for sickle cell disease. J Natl Med Assoc.

[5] Werner EM (2013). NHLBI activities in hemoglobinopathies. NICHD Newborn Screening Translational Research Network Meeting.

[6] Treadwell MT, Hassell KL, Levine RE, Keller SD: **Adult Sickle Cell Quality of Life Measurement Information System (ASCQ-Me): Conceptual model based on review of the literature and formative research.** *Clin J Pain* 2013, [Epub ahead of print]. 10.1097/AJP.0000000000000054 PMC4993284 24300219

[7] Keller SD, Levine RE (2011). The grounded item technique for developing content valid questions.[abstract]. Qual Life Res.

[8] Flanagan JC (1954). The critical incident technique. Psych Bull.

[9] Stewart AL, Ware JE (1992). Measuring Functioning and Well-being: The Medical Outcome Study Approach.

[10] DeWalt DA, Rothrock N, Yount S, Stone AA, PROMIS Cooperative Group (2007). Evaluation of item candidates: The PROMIS qualitative item review. Med Care.

[11] Reise SP, Yu J (1990). Parameter recovery in the graded response model using MULTILOG. J Educ Meas.

[12] Bender MA, Hobbs W, Pagon RA, Bird TC, Dolan CR, Stephens K (1993–2003). Sickle Cell Disease. Gene Reviews [Internet].

[13] Steinberg MH (2008). Sickle cell anemia, the first molecular disease: overview of molecular etiology, pathophysiology, and therapeutic approaches. Sci World J.

[14] Steinberg MH (2005). Predicting clinical severity in sickle cell anaemia. Br J Haematol.

[15] Sebastiani P, Nolan VG, Baldwin CT, Abad-Grau MM, Wang L, Adewoye AH, McMahon LC, Farrer LA, Taylor JG, Kato GJ, Gladwin MT, Steinberg MH (2007). A network model to predict the risk of death in sickle cell disease. Blood.

[16] Akinola NO, Bolarinwa RA, Faponle AF (2009). The import of abdominal pain in adults with sickle cell disorder. West Afr J Med.

[17] Ballas SK (2010). Sickle cell disease: current clinical management. Semin Hematol.

[18] Sebastiani P, Solovieff N, Hartley SW, Milton JN, Riva A, Dworkis DA, Melista E, Klings ES, Garrett ME, Telen MJ, Ashley-Koch A, Baldwin CT, Steinberg MH (2010). Genetic modifiers of the severity of sickle cell anemia identified through a genome-wide association study. Am J Hematol.

[19] Steinberg MH, Adewoye AH (2006). Modifier genes and sickle cell anemia. Curr Opin Hematol.

[20] Mayer ML, Konrad TR, Dvorak CC (2003). Hospital resource utilization among patients with sickle cell disease. J Health Care Poor Underserved.

[21] Loureiro MM, Rozenfeld S, Sá Carvalho M, Portugal RD (2009). Factors associated with hospital readmission in sickle cell disease. BMC Blood Disord.

[22] Aisiku IP, Smith WR, McClish DK, Levenson JL, Penberthy LT, Roseff SD, Bovbjerg VE, Roberts JD (2009). Comparisons of high versus low emergency department utilizers in sickle cell disease. Ann Emerg Med.

[23] Audard V, Homs S, Habibi A, Galacteros F, Bartolucci P, Godeau B, Renaud B, Levy Y, Grimbert P, Lang P, Brun-Buisson C, Brochard L, Schortgen F, Maitre B, Mekontso Dessap A (2010). Acute kidney injury in sickle patients with painful crisis or acute chest syndrome and its relation to pulmonary hypertension. Nephrol Dial Transplant.

[24] Frei-Jones MJ, Field JJ, DeBaun MR (2009). Risk factors for hospital readmission within 30 days: a new quality measure for children with sickle cell disease. Pediatr Blood Cancer.

[25] Carroll CP, Haywood C, Fagan P, Lanzkron S (2009). The course and correlates of high hospital utilization in sickle cell disease: Evidence from a large, urban Medicaid managed care organization. Am J Hematol.

[26] Smith WR, Penberthy LT, Bovbjerg VE, McClish DK, Roberts JD, Dahman B, Aisiku IP, Levenson JL, Roseff SD (2008). Daily assessment of pain in adults with sickle cell disease. Ann Intern Med.

[27] Dampier C, Setty BN, Eggleston B, Brodecki D, O'neal P, Stuart M (2004). Vaso-occlusion in children with sickle cell disease: clinical characteristics and biologic correlates. J Pediatr Hematol Oncol.

[28] Vick LR, Gosche JR, Islam S (2009). Partial splenectomy prevents splenic sequestration crises in sickle cell disease. J Pediatr Surg.

[29] Halabi-Tawil M, Lionnet F, Girot R, Bachmeyer C, Lévy PP, Aractingi S (2008). Sickle cell leg ulcers: a frequently disabling complication and a marker of severity. Br J Dermatol.

[30] Nolan VG, Adewoye A, Baldwin C, Wang L, Ma Q, Wyszynski DF, Farrell JJ, Sebastiani P, Farrer LA, Steinberg MH (2006). Sickle cell leg ulcers: associations with haemolysis and SNPs in Klotho, TEK and genes of the TGF-beta/BMP pathway. Br J Haematol.

[31] Klings ES, Wyszynski DF, Nolan VG, Steinberg MH (2006). Abnormal pulmonary function in adults with sickle cell anemia. Am J Respir Crit Care Med.

[32] Gill FM, Sleeper LA, Weiner SJ, Brown AK, Bellevue R, Grover R, Pegelow CH, Vichinsky E (1995). Clinical events in the first decade in a cohort of infants with sickle cell disease. Cooperative study of sickle cell disease. Blood.

[33] Van der Linden WT, Van K-S (2003). Using response times to detect aberrant responses in computerized adaptive testing. Psychometrika.

[34] Cheng L, Cumber S, Dumas C, Winter R, Nguyen KM, Nieman LZ (2003). Health related quality of life in pregeriatric patients with chronic diseases at urban, public supported clinics. Health Qual Life Outcomes.

[35] Wensing M, Vingerhoets E, Grol R (2001). Functional status, health problems, age and comorbidity in primary care patients. Qual Life Res.

[36] Michelson H, Bolund C, Brandberg Y (2000). Multiple chronic health problems are negatively associated with health related quality of life (HRQoL) irrespective of age. Qual Life Res.

[37] Meyer HH (1951). Methods for scoring a check-list type rating scale. J Appl Psychol.

[38] Bland AC, Kreiter CD, Gordon JA (2005). The psychometric properties of five scoring methods applied to the script concordance test. Acad Med.

[39] Hu L, Bentler PM (1999). Cutoff criteria for fit indexes in covariance structure analysis: Conventional criteria versus new alternatives. Struct Equ Model.

[40] Suhr DD: **Exploratory or confirmatory factor analysis?** *SUGI Proc* 2006, Paper 200–31.

[41] Horn JL (1965). A rationale and a test for the number of factors in factor analysis. Psychometrika.

[42] Cattell RB (1966). The scree test for the number of factors. Multivar Behav Res.

[43] Thurstone LL (1935). The vectors of the mind: multiple factor analysis for the isolation of primary traits.

[44] Hayashi T, Hays RD (1987). A microcomputer program for analyzing multitrait-multimethod matrices. Behav Res Methods Instrum Comput.

[45] Ware JE, Harris WJ, Gandek B, Rogers BW, Reese PR (1997). MAP-R for Windows: Multitrait/Multi-item Analysis Program - Revised User's Guide.

[46] Howard KI, Forehand GG (1962). A method for correcting item-total correlations for the effect of relevant item inclusion. Ed Psych Meas.

[47] Reise SP, Morizot J, Hays RD (2007). The role of the bifactor model in resolving dimensionality issues in health outcomes measures. Qual Life Res.

[48] Samejima F (1969). Estimation of latent ability using a response pattern of graded scores. Psychometrika Monograph, 17.

[49] Thissen D, Weiss DJ (2009). The MEDPRO project: An SBIR project for a comprehensive IRT and CAT software system—IRT software. Proceedings of the 2009 GMAC Conference on Computerized Adaptive Testing.

[50] Harrison DA (1986). Robustness of IRT parameter estimation to violations of the unidimensionality assumption. J Educ Stat.

[51] Embretson SE, Reise SP (2000). Item Response Theory for Psychologists.

[52] Woods CM (2009). Empirical selection of anchors for tests of differential item functioning. Appl Psychol Meas.

[53] Lord FM, Poortinga YH (1977). A Study of Item Bias, using item Characteristic Curve Theory. Basic Problems in Cross-Cultural Psychology.

[54] Lord FM (1980). Applications of Item Response Theory to Practical Testing Problems.

[55] Langer MM (2008). A reexamination of Lord’s Wald test for differential item functioning using item response theory and modern error estimation (Doctoral Dissertation).

[56] Bock RD, Aitkin M (1981). Marginal maximum likelihood estimation of item parameters: Application of an EM algorithm. Psychometrika.

[57] National Center for Health Statistics (2012). Health, United States, 2011: With Special Feature on Socioeconomic Status and Health.

[58] Platt OS, Thorington BD, Brambilla DJ (1991). Pain in sickle cell disease – rates and risk factors. N Engl J Med.

[59] Ballas SK, Lusardi M (2005). Hospital readmission for adult acute sickle cell painful episodes: frequency, etiology, and prognostic significance. Am J Hematol.

[60] Yusuf HR, Atrash HK, Grosse SK, Parker CS, Grant AM (2010). Emergency department visits made by patients with sickle cell disease: A descriptive Study, 1999–2007. Am J Prev Med.

[61] Hays RD, Bjorner JB, Revicki DA, Sprintzer KL, Cella D (2009). Development of physical and mental health summary scores from the patient-reported outcomes measurement information system (PROMIS) global items. Qual Life Res.

[62] Cronbach LJ (1951). Coefficient alpha and the internal structure of tests. Psychometrika.

[63] Tomarken AJ, Waller NG (2005). Structural equation modeling: Strengths, limitations, and misconceptions. Annu Rev Clin Psychol.

[64] Cook KF, Kallen MA, Amtmann D (2009). Having a fit: impact of number of items and distribution of data on traditional criteria for assessing IRT’s unidimensionality assumption. Qual Life Res.

[65] Kenny DA (2012). Measuring Model Fit.

[66] Bayliss EA, Ellis JL, Steiner JF (2005). Subjective assessments of comorbidity correlate with quality of life health outcomes: Initial validation of a comorbidity assessment instrument. Health Qual Life Outcomes.

[67] *Registry and Surveillance System for Hemoglobinopathies Pilot Project (RuSH).* [http://www.cdc.gov/ncbddd/sicklecell]

[68] Dampier C, LeBeau P, Rhee S, Lieff S, Kesler K, Ballas S, Rogers Z, Wang W, Comprehensive Sickle Cell Centers (CSCC) Clinical Trial Consortium (CTC) Site Investigators (2011). Health-related quality of life in adults with sickle cell disease (SCD): a report from the comprehensive sickle cell centers clinical trial consortium. Am J Hematol.

[69] Levenson JL, McClish DK, Dahman BA, Bovbjerg VE, De ACitero V, Penberthy LT, Aisiku IP, Roberts JD, Roseff SD, Smith WR (2008). Depression and anxiety in adults with sickle cell disease: the PiSCES project. Psychosom Med.

[70] McClish DK, Levenson JL, Penberthy LT, Roseff SD, Bovbjerg VE, Roberts JD, Aisiku IP, Smith WR (2006). Gender differences in pain and healthcare utilization for adult sickle cell patients: The PiSCES Project. J Womens Health (Larchmt).

[71] Reeve BB, Hays RD, Bjorner JB, Cook KF, Crane PK, Teresi JA, Thissen D, Revicki DA, Weiss DJ, Hambleton RK, Liu H, Gershon R, Reise SP, Lai JS, Cella D, PROMIS Cooperative Group (2007). Psychometric evaluation and calibration of health-related quality of life item banks: Plans for the Patient-Reported Outcomes Measurement Information System (PROMIS). Med Care.

